# Altered Neural Processing of Reward and Punishment in Women With Methamphetamine Use Disorder

**DOI:** 10.3389/fpsyt.2021.692266

**Published:** 2021-10-13

**Authors:** Shuguang Wei, Zhaoxia Xue, Wujun Sun, Jie Han, Haiyan Wu, Xun Liu

**Affiliations:** ^1^Department of Psychology, College of Education, Hebei Normal University, Shijiazhuang, China; ^2^Department of Applied Psychology, College of Humanities and Social Sciences, Shanxi Medical University, Taiyuan, China; ^3^Faculty of Education, Henan Normal University, Xinxiang, China; ^4^Hebei Female Drug Rehabilitation Center, Shijiazhuang, China; ^5^Centre for Cognitive and Brain Sciences and Department of Psychology, University of Macau, Macao, Macao, SAR China; ^6^Key Laboratory of Behavioral Science, Institute of Psychology, Chinese Academy of Sciences, Beijing, China; ^7^Department of Psychology, University of Chinese Academy of Sciences, Beijing, China

**Keywords:** methamphetamine (MA) use disorder, reward processing, punishment processing, Cue-P3, stimulus-preceding negativity (SPN), feedback-related negativity (FRN), feedback P3 (FB-P3)

## Abstract

It has been suggested that the altered function of reward and punishment is an important vulnerability factor leading to the development of drug use disorders. Previous studies have identified evidence of neurophysiological dysfunction in the reward process of individuals with substance use disorders. To date, only a few event-related potential (ERP) studies have examined the neural basis of reward and punishment processing in women with methamphetamine (MA) use disorders. The current ERP research aims to investigate the neurophysiological mechanisms of reward and punishment in women with MA use disorder using a monetary incentive delay task. Nineteen women with MA use disorder (MA group) and 20 healthy controls (HC group) were recruited in this study. The behavioral data showed that the reaction time (RT) was faster and the response accuracy (ACC) was higher for the potential reward and punishment conditions compared to neutral conditions. During the monetary incentive anticipation stage, the Cue-P3, and stimulus-preceding negativity (SPN) were larger in the MA group than in the HC group. The SPN under the potential reward condition was larger than that under the neutral condition in the MA group but not in the HC group. During the monetary incentive consummation stage, the feedback-related negativity and feedback P3 (FB-P3) following positive feedback were significantly larger than negative feedback in the potential reward condition for the HC group, but not for the MA group. However, the FB-P3 following negative feedback was significantly larger than positive feedback in the potential punishment condition for the MA group, but not the HC group. The results suggest that women with MUD have stronger expectations of generic reward and stronger response of generic harm avoidance, which could be targeted in designing interventions for women with MA use disorder.

## Introduction

Substance use disorder (SUD) is characterized by chronic relapse, compulsive drug use, and loss of control over drug-taking behavior despite adverse consequences (1). Methamphetamine (MA) is the second most widely used illegal drug worldwide (2), and the use of MA in China has exceeded heroin use as the most widely abused drug in recent years (3). MA can stimulate the rewarding system of the brain and has highly reinforcing effects that lead to abuse and dependence. Chronic MA abuse is associated with significant neurological damage and psychiatric impairment in the cognitive, intellectual, and affective domains (4–6). However, the neural correlates in individuals with MA use disorder (MUD) are not well-understood.

The outcomes of a particular behavior, choice, or environment have a significant influence on motivation and decision-making. These results, whether positive (rewards) or negative (punishments), can strongly influence an individual's behavior (7). Rewards can be defined as stimuli that an organism tries to obtain, while punishments are stimuli that an organism tries to avoid. By definition, reinforcement is a stimulus that can increase the frequency of a behavior, and positive reinforcement and rewards are generally considered to be synonymous. Negative reinforcement refers to a decrease in aversive stimuli leading to an increase in individual behavioral responses. Punishment consists of the presentation of an aversive stimulus or the removal of an appetitive stimulus. Researchers have suggested that the brain mechanisms of positive and negative reinforcement have been considered key to the etiology and maintenance of the pathophysiology of addiction (8–11). Instead of seeking rewards and avoiding punishment, addicts are driven to seek special rewards to compromise other needs or attribute rewards to maladaptive behaviors (12). Therefore, understanding the neural processing mechanisms of rewards and punishments is very important for understanding the brainpower of substance users.

Various experimental paradigms have been used to explain reinforcement processing for individuals with and without mental health disorders. One of the most well-established paradigms is the monetary incentive delay (MID) task (13). A typical trial requires a quick response to a target following a cue-signaling contingency for that trial. Performance-specific feedback is delivered based on the response. The MID task has been used in many functional magnetic resonance imaging (fMRI) studies to effectively delineate the dynamics of brain activity in reward processing [i.e., anticipation and consummation; for a meta-analysis, see (14)]. Individuals with SUD show enhanced reward-related responses to drug-related cues [see meta-analyses (15, 16)]. However, there are still inconsistencies regarding the response to non-drug rewards in the anticipatory and consummatory stages in SUD. Using fMRI, researchers identified that regular smokers show reduced ventral striatum (VS) recruitment in response to monetary anticipatory cues or monetary notifications compared to controls (17–19). However, research on the use of other substances is more inconsistent; several studies found no decrease in VS recruitment by non-drug reward cues or delivery in substance users (20, 21). The results also varied as a function of whether the anticipatory or consummatory component was emphasized. Schmidt et al. (22) found that individuals with gambling disorder showed greater left orbitofrontal cortex and VS activity to erotic relative to monetary reward anticipation compared to healthy volunteers, but generally stronger activity in the VS, ventromedial and dorsolateral prefrontal cortex, and anterior cingulate cortex to both erotic and monetary rewards relative to healthy volunteers. Using an image-based meta-analysis, a systematic literature review (23) concluded that substance users show decreased striatal activation during monetary reward anticipation and increased VS activation during monetary reward consummation.

In addition to the neural mechanisms underlying reward processing, drug-seeking behavior is also a function of punishment processing (9). In the development of addiction, the negative effects of drug withdrawal have become the main motivation for drug use. That is, negative reinforcement plays an important role in the maintenance of addiction. In drug addiction, the withdrawal response brought on by an individual ceasing drug use and that individual's negative emotional state are important reasons for their drug-taking behavior. Compared to reward processing, the neural bases of punishment processing remain largely unexamined in substance users. An fMRI study investigating responses to monetary gains and losses demonstrated that individuals with MUD exhibited less response in the VS to loss anticipation than controls, but more response in the caudate to loss outcomes than to gain outcomes (24). However, other studies indicated that substance users have a blunted response to punishment. For example, one study illustrated that cocaine-dependent participants showed diminished behavioral punishment sensitivity, which was associated with significant deactivation in the dorsal anterior cingulate cortex, right insula, and right prefrontal regions (25). Romanczuk-Seiferth et al. (26) investigated the neural correlates of loss processing in pathological gamblers compared with alcohol-dependent patients and healthy controls, and found that pathological gamblers showed increased activity in the right VS during loss anticipation compared with controls and alcohol-dependent patients. Moreover, pathological gamblers showed decreased activation in the right VS and right medial prefrontal cortex during successful loss avoidance compared with controls. Other studies also confirmed that smokers have a lower error-correction rate and are less sensitive to punishment (27, 28). Compared to reward processing, significant work is required to link punishment processing to specific neural mechanisms in individuals with SUD.

Studies using fMRI demonstrated dissociable patterns of activation in response to monetary outcomes (29, 30). As a complement to neuroimaging research, event-related potentials (ERPs) provide superior millisecond-by-millisecond temporal resolution, thus enabling a full characterization of reward processing. The MID task also allows for exploration of the neurophysiological correlates of reward and punishment processing in one experimental paradigm (31, 32). According to the framework of the MID, several candidate ERP components may be relevant to different stages of reward and punishment processing. The reinforcement anticipatory stage is associated with three ERP components: Cue-P3, contingent negative variation (CNV), and stimulus-preceding negativity (SPN). First, Cue-P3 is involved in the attention allocation of incentive-contingent cues. Cue-P3 is a late positive-going component that peaks between 300 and 600 ms post-stimulus at centroparietal sites. Cue-P3 is generally more positive for salient, task-relevant, or unexpected stimuli (33), and it is increased for incentive vs. neutral cues in MID tasks (31, 32). Second, the CNV is a slow negative-going potential that occurs between a warning stimulus (cue) and an imperative stimulus (target) (34), and can reflect anticipatory attention, motivation, and motor preparation (32). The third sub-stage within reinforcement anticipation is the interval following the motor reaction, and in anticipation of the outcome present, which should elicit an SPN. Compared to CNV, SPN reflects pure anticipatory processing due to the exclusion of motor preparation (35).

Regarding the consummatory stage, feedback-related negativity (FRN) and feedback P3 (FB-P3) are the relevant ERP components. FRN is typically defined as a negative-going component that peaks at ~250 ms after outcome onset. It is thought to encode the reward prediction error (the difference between predicted and obtained outcomes) when feedback is better or worse than expected (36). However, more recent research supports the view that FRN is driven by reward delivery (37). FB-P3 is a centroparietal positive-going component approximately peaking at 300–600 ms following feedback. FB-P3 involves the classification of important attentionally driven information related to outcomes, such as context updating and integration of the contents of working memory to maximize future rewards (38). Additionally, FB-P3 may reflect affective processes by signaling the motivational salience of reward feedback (39).

Using ERPs, Morie et al. (40) found that cocaine users demonstrated increased neural response to monetary incentive cues indexed by cue-related negativities and CNV; however, Zhao et al. (41) demonstrated that heroin users showed blunted neural response indexed by disrupted SPN during the reward anticipation stage. In the reward consummatory stage, many studies found that individuals with cocaine and alcohol use disorder showed blunted sensitivity to monetary reward outcomes indexed by decreased FRN and FB-P3 (42–45). However, Zhao et al. (41) found that heroin users showed enhanced neural response to monetary feedback indexed by FRN. In our previous research, using a simple gamble task in a separate MUD group, we found an enhanced neural response to monetary cues and feedback indexed by SPN, FRN, and FB-P3 (46). With the ERP and fMRI studies taken together, the contradictory evidence as to whether individuals with SUD show an enhanced or blunted neural response to monetary rewards calls for more detailed research in this field.

Much of the initial research on SUD came from the studies conducted with male substance users. However, in recent years, some studies have found that, compared with male substance users, female substance users have more sensitive psychomotor-related responses to addictive substances (47), and can more easily transition from recreational use to SUD (48–50). Therefore, exploring the female-specific MA use behaviors is crucial to the development of appropriate MA use prevention and treatment strategies.

The current ERP study aimed to investigate the neurophysiological mechanisms underlying anticipation and consummation of reward and punishment in women with MUD. Therefore, we used the MID task, which included a separate punishment and reward condition. We focused on Cue-P3, CNV, and SPN to examine anticipatory processes and on FRN and FB-P3 to examine consummatory processes. Based on our previous ERP study on women with MUD indicating enhanced neural responsivity to reward, we expected enhanced ERP components (Cue-P3/CNV/SPN/FRN/FB-P3) during reward anticipation and consummation in women with MUD compared to controls. Regarding punishment consummation, as our prior study showed that women with MUD made more risky choices following a loss outcome in a previous trial, we expected blunted ERP components (Cue-P3/CNV/SPN/FB-P3/FRN) during punishment anticipation and consummation in women with MUD compared to controls.

## Materials and Methods

### Participants

Nineteen female MA users (age = 25 ± 4.41 years; drug experience = 23.42 ± 10.05 months; abstinence duration = 14.53 ± 3.84 months) participated in the study as the experimental group (MA group). They were patients from an addiction rehabilitation center in Hebei Province, China. All patients were subjected to a 24-month compulsory isolation treatment, during which they were unable to use cigarettes, alcohol, or addictive substances. Twenty healthy female participants without a history of substance use (age = 27.05 ± 4.75 years) were selected for the healthy control group (HC group). They were recruited using advertisements on the Internet and *via* word-of-mouth from the same geographic area.

The inclusion criteria for the MA group were as follows: (1) a history of MA use corresponding to the diagnosis of stimulant addiction disorder using Diagnostic and Statistical Manual of Mental Disorders Fifth Edition (DSM-5) (51); (2) a drug withdrawal period from 3 to 24 months before the date of screening. The selection criteria for the HC group were similar to the selection criteria for the MA group. In the HC group, all participants reported having no history or current use of illegal drugs. The exclusion criteria were as follows: (1) a history of using other kinds of drugs (e.g., heroin, cocaine), (2) a history of brain injury leading to loss of awareness of more than 30 min, (3) current or a history of brain pathology, and (4) a history of using any psychotropic drug within 2 months of this study registration.

The screening process was similar to that used in a previous study (40). After entering the test room, all participants were asked about their drug use time, abstinence time, cumulative drug dosage, the number of cigarettes consumed, and alcohol usage per day for the month before their treatment. Furthermore, all participants were asked to complete the Barratt Impulsiveness Scale Version 11 (BIS-11) (52) and the Sensation Seeking Scale Version V (SSS-V) (53). Each received a base payment of ¥40 for participating and a bonus of up to ¥10 based on their performance in the MID task. All participants were right-handed and had normal or corrected-to-normal visual acuity. Written informed consent was obtained from all participants. The study was conducted under the Declaration of Helsinki and approved by the ethical review board of the Institute of Psychology of the Chinese Academy of Science.

### ERP Task—The MID Task

The participants completed the test in a sound-attenuating room. At the start of the experiment, participants were informed that they needed to respond as quickly as possible, and their performance was related to the bonus.

**Figure 1:**
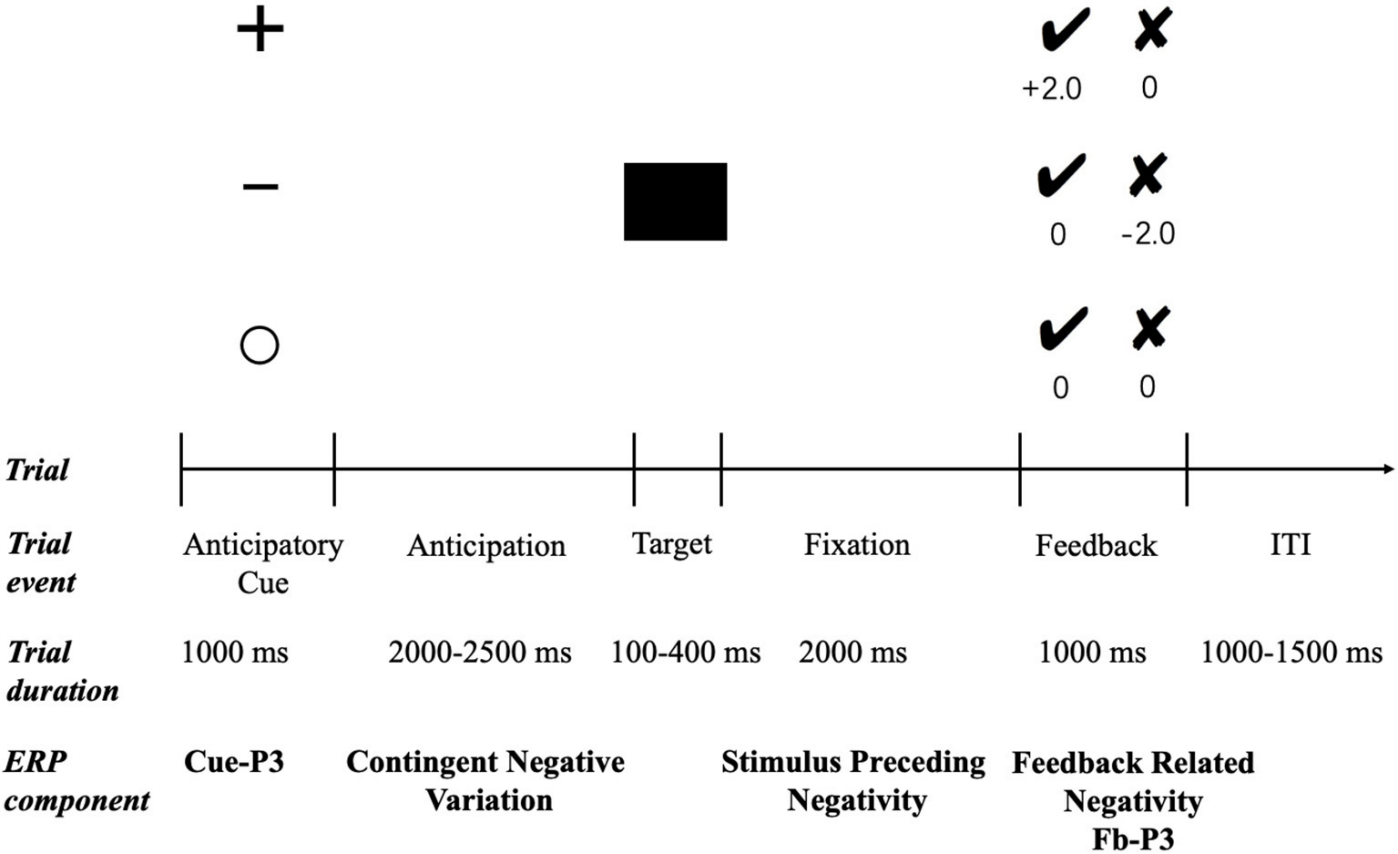
Trial structure and timeline of the MID task. ITI, intertrial interval.

All participants were asked to complete a modified version of the MID (32, 54) (see Figure 1). In each trial, one of the three cues depicting the monetary contingency for that trial was presented for 1,000 ms. The plus sign indicated a potential monetary reward (potential reward condition), the minus sign indicated a potential monetary punishment (potential punishment condition), and the empty circle indicated that no monetary outcome would be delivered irrespective of performance (neutral condition). Thus, following a jittered interstimulus interval (ISI; 2,000–2,500 ms), a black square was presented as the target stimulus, and the participants were instructed to respond by pressing a button as quickly as possible. The duration of the target presentation was set to 250 ms initially and then was adapted between 100 and 400 ms according to participants' response times. Specifically, the target duration was decreased by 25 ms after a successful response (i.e., pressing the button during target presentation) and increased by 25 ms after an unsuccessful response (i.e., pressing the button either before or after target presentation). This staircase process resulted in a success rate of ~50% for all three conditions. Following another ISI (2,000 ms), the performance feedback was presented for 1,000 ms. Positive feedback was indicated by a black tick and negative feedback by a black cross. In potential reward trials, the tick feedback signaled a win of ¥2, whereas the cross feedback signaled a win of ¥0. In potential punishment trials, the tick feedback indicated a loss of ¥0, whereas the cross feedback signaled a loss of ¥2. In neutral trials, both crosses and ticks led to ¥0. All trials in the three conditions were randomly presented during the experiment. The task consisted of three blocks, 240 trials in total (80 trials for each condition), and there was a short break between blocks. Before the formal experimentation, there was a practice session to familiarize participants with the task.

### Psychophysiological Recording and Data Analysis

Continuous scalp electroencephalographic (EEG) activity was recorded using an electrode cap with 64 electrodes according to a modified expanded 10–20 system (Brain Products Company, Munich, Germany). The signals were recorded online using the reference electrode FCz and the ground electrode AFz. An electrode was placed ~2 cm below the right eye to record vertical electrooculogram. The impedance between all the electrodes and the scalp was <5 kΩ.

EEGLAB (55) and ERPLAB (56) were used to analyze the EEG data. All signals were re-referenced to the bilateral mastoid average (TP9/10) and low-pass filtered of 30 Hz (roll-off 6 dB/octave). For the Cue-P3 and CNV, the EEG data were segmented from −200 to 3,000 ms relative to cue onset, with −200 to 0 as the baseline. For the SPN, the EEG data are segmented from −2,000 to 200 ms relative to feedback onset, with −1,900 to −1,700 ms as the baseline. For the FRN and FB-P3, the EEG data were segmented from −200 to 1,000 ms relative to feedback onset with the activity from −200 to 0 serving as the baseline. Epochs containing artifacts outside −80 to 80 μV were eliminated. Independent component analysis (ICA) (runica) was performed. Subsequently, eye blinking and movement artifacts were selected and removed manually. Thus, the epochs in the same condition were averaged for each participant. In the anticipatory stage, there were 73.58 ± 5.82 (72.45 ± 8.18), 72.79 ± 5.52 (71.1 ± 9.67), and 72.79 ± 5.83 (70.55 ± 11.62) artifact-free trials obtained for the monetary reward, monetary punishment, and neutral conditions in the MA group (HC group). In the consummatory stage, there were 44.79 ± 4.85 (42.40 ± 5.31), 35.47 ± 4.36 (37.30 ± 4.99), 42.79 ± 3.41 (41.75 ± 4.94), 36.53 ± 3.64 (37.90 ± 5.07), 33.36 ± 5.73 (35.75 ± 5.97), and 47.05 ± 5.33 (44.65 ± 6.08) artifact-free trials obtained for the hit and miss of the monetary reward, monetary punishment, and neutral condition in the MA group (HC group), respectively.

Following a previous study, ERP components were quantified using a region-of-interest (ROI) approach (40, 41). Cue-P3 and FB-P3 were measured as the mean amplitude from 300 to 450 ms post-cue or feedback onset over a centroparietal ROI (C1, Cz, C2, CP1, CPz, CP2, P1, Pz, and P2) and the CNV from 2,800 to 3,000 ms post cue onset over the frontal–central ROI (F1, F2, Fz, FC1, FC2, FCz, C1, C2, and Cz). Given a plateau-shaped distribution with a right hemisphere dominance (28), in this study, the SPN was measured as the mean amplitude from −200 to 0 ms before feedback onset over the right frontotemporal ROI (F8, FT8, T8, F6, FC6, C6, F4, FC4, and C4). The FRN was measured as the mean amplitude from 200 to 300 ms post-feedback onset over the frontocentral ROI (F1, F2, Fz, FC1, FC2, FCz, C1, C2, and Cz).

### Statistical Analysis

For the demographic characteristics, independent samples *t*-tests were used to compare group differences (MA vs. HC). For behavioral data from the MID task, a 2 (group: MA vs. HC) ×3 (incentive: potential reward vs. potential punishment vs. neutral) repeated-measures ANOVA was performed on the response time (RT) and the response accuracy (ACC), where the group was a between-subjects variable and the incentive was a within-subjects variable.

Separate repeated-measures ANOVAs were used for all ERP data. A 2 (group: MA vs. HC) ×3 (incentive: potential reward vs. potential punishment vs. neutral) repeated-measures ANOVA was performed on the Cue-P3, CNV, and SPN data, with group as a between-subjects variable and incentive as a within-subjects variable. For the FRN and FB-P3, a 2 (groups: MA vs. HC) ×3 (incentive: potential reward vs. potential punishment vs. neutral) ×2 (feedback: positive vs. negative) repeated-measures ANOVA was performed, with group as a between-subjects variable, and incentive and feedback as within-subjects variables. When significant interaction effects were indicated, further simple effect analyses were performed. The Greenhouse–Geisser correction was applied when detecting violations of sphericity, and statistical significance was set at *p* < 0.05. The measures of the proportion between the variance of one experimental factor and the total variance were reported in partial eta squared (η_*p*_^2^).

## Results

### Behavioral Data

Table 1 shows the group differences regarding drug use time, abstinence time, cumulative drug dosage, the number of cigarettes consumed, and alcohol usage per day for 1 month prior to treatment. There were no significant differences between the two groups in age or education (*p*s > 0.05). The MA group scored significantly higher than the HC group on the subscales for motor impulsiveness and non-planning impulsiveness (*p*s < 0.05). Similarly, the MA group had significantly higher scores on the Sensation Seeking Scale and its subscales of disinhibition and experience seeking compared with the HC group (*p*s <0.01).

**Table 1:** Sample characteristics (M ± SD).

	**HC group** **(*n* = 20)**	**MA group** **(*n* =19)**	***p*-values**
Age (years)	27.05 ± 4.75	25 ± 4.41	0.17
Education (years)	9.15 ± 0.67	8.82 ± 2.16	0.51
Drug experience (months)	–	23.42 ± 10.05	
Abstinence time (months)	–	14.53 ± 3.84	
Methamphetamine use, lifetime (g)	–	266.13 ± 407.42	
Number of cigarettes per day	–	8 ± 8.27	
Alcohol use per day (g)	–	23.03 ± 63.23	
BIS-11	63.75 ± 11.02	69.84 ± 9.83	0.08
Attentional impulsiveness	18.3 ± 5.18	17.07 ± 2.99	0.37
Motor impulsiveness	20.56 ± 3.97	23.28 ± 3.78	<0.05^*^
Non-planning impulsiveness	25.27 ± 5.55	29.49 ± 5.51	<0.05^*^
SSS-V	12.7 ± 4.07	17.32 ± 4.85	<0.01^**^
Disinhibition	2.35 ± 2	4.16 ± 2.54	<0.01^**^
Experience seeking	3.6 ± 1.9	5.23 ± 1.65	<0.01^**^
Thrill and adventure seeking	4.5 ± 2.97	5.39 ± 2.19	0.3
Boredom susceptibility	2.25 ± 1.4	2.56 ± 1.4	0.5

*BIS-11, Barratt Impulsiveness Scale, Version 11; SSS-V, Sensation Seeking Scale Form V.*

* *p < 0.05,*

** *p < 0.01*.

Descriptive behavioral data are presented in Table 2. RTs were analyzed using a 2 × 3 ANOVA. There was a significant main effect of group [*F*_(1, 37)_ = 4.93, *p* < 0.05, and η_*p*_^2^ = 0.12]. RTs in the MA group (202.16 ms) were significantly faster than those in the HC group (226.94 ms). An independent *t*-test on three incentive conditions showed that RTs in the MA group were significantly fast than those in the HC group in the potential reward [*t*_(37)_ = 2.25, *p* < 0.05] and neutral conditions [*t*_(37)_ = 2.33, *p* < 0.05]; the group difference in RTs was marginally significant in the potential punishment condition [*t*_(37)_ = 1.96, *p* = 0.06]. The main effect of the incentive condition was significant [*F*_(2, 74)_ = 6.69, *p* < 0.01, and η_*p*_^2^ = 0.15]. Pairwise comparisons revealed that the RTs were faster for potential reward (211.82 ms) and potential punishment trials (213.46 ms) compared to neutral trials (218.32 ms, *p*s < 0.05). There were no significant differences between the potential reward and potential punishment trials (*p* > 0.05). The interaction effect of group and incentive condition was not significant [*F*_(1, 37)_ = 0.96, *p* = 0.34, and η_*p*_^2^ = 0.03].

**Table 2:** Group means and standard deviations (in brackets) of reaction times (RTs) and response accuracy (ACC) for MA and HC group.

	**HC group** **(*n* = 20)**	**MA group** **(*n* = 19)**
RTs in potential monetary reward trials	224.41 (43.68)	199.23 (22.05)
RTs in potential monetary punishment trials	224.5 (42.95)	202.41 (24.4)
RTs in neutral trials	231.91 (44.01)	204.73 (26.24)
ACC in potential monetary reward trials	0.53 (0.07)	0.56 (0.06)
ACC in potential monetary punishment trials	0.51 (0.1)	0.54 (0.04)
ACC in neutral trials	0.43 (0.09)	0.41 (0.08)

The ACC was subjected to a 2 × 3 ANOVA. There was a significant main effect of incentive condition [*F*_(2, 74)_ = 27.46, *p* < 0.001, and η_*p*_^2^ = 0.43]. Pairwise comparisons revealed that the ACC was higher for the potential reward (0.54) and punishment trials (0.53) compared to neutral trials (0.42, *p*s < 0.001). There were no significant differences between the potential reward and potential punishment trials (*p* > 0.05). The main effect of group [*F*_(1, 37)_ = 1.25, *p* = 0.27, and η_*p*_^2^ = 0.03] and the interaction effect of group and incentive condition were not significant [*F*_(2, 74)_ = 1.35, *p* = 0.26, and η_*p*_^2^ = 0.04]. Thus, these results indicate incentive-related accuracy and speed in the MID task.

### Electrophysiological Data

#### Anticipatory ERPs

##### Cue-P3

**Figure 2:**
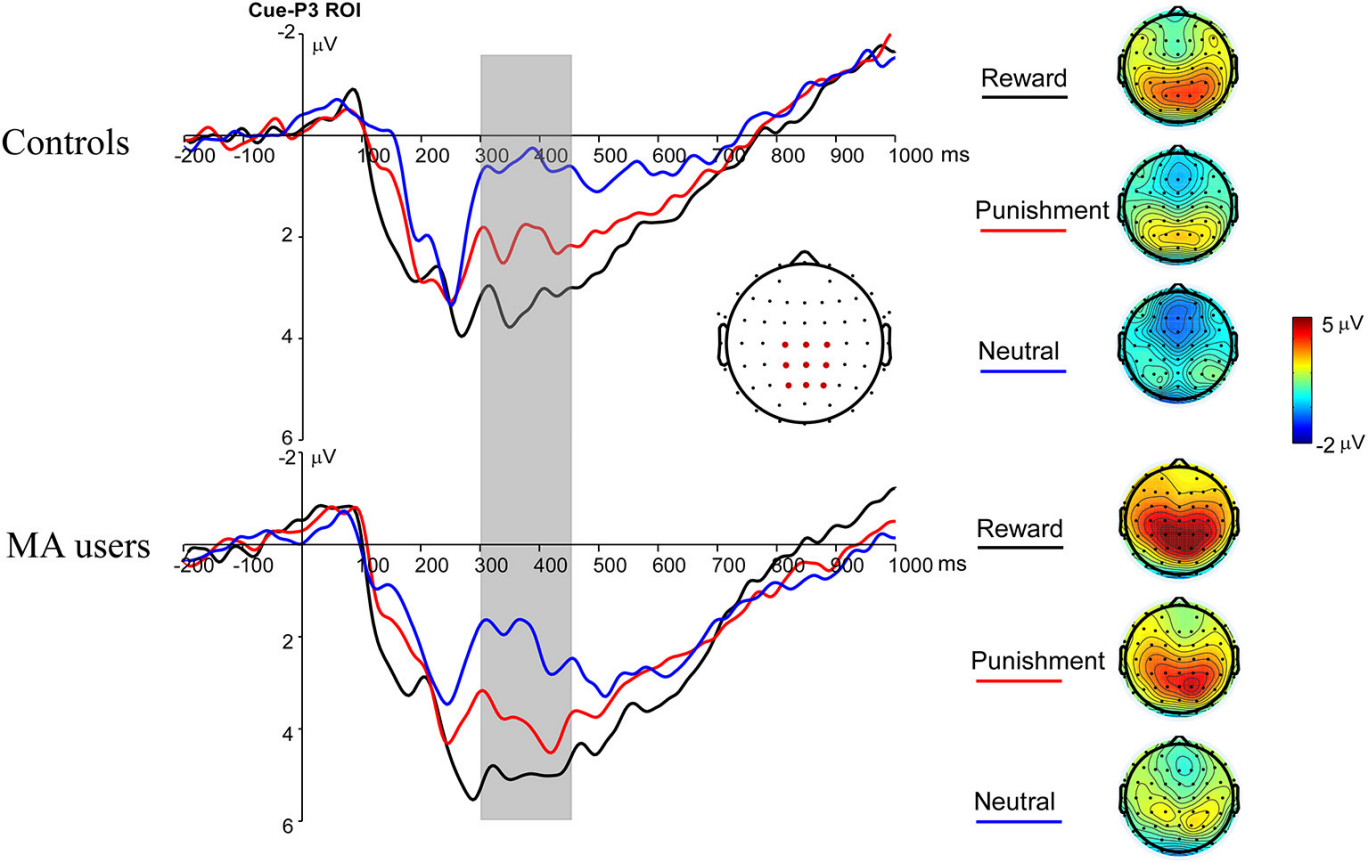
Cue-P3 waveforms after cue presentation for the MA and HC group over centroparietal ROI (C1, Cz, C2, CP1, CPz, CP2, P1, Pz, and P2) (left); topographic maps of the Cue-P3 during 300–450 ms after cue presentation (right).

A 2 × 3 ANOVA was performed on the Cue-P3 data. There was a significant main effect of incentive condition [*F*_(2, 74)_ = 18.34, *p* < 0.001, and η_*p*_^2^ = 0.33]. Pairwise comparisons revealed that the Cue-P3 was more positive for potential reward (3.66 μV) and punishment trials (2.84 μV) compared to neutral trials (1.81 μV, *p*s < 0.001), and marginally positive for reward trials compared to punishment trials (*p* = 0.07). The main effect of group was also significant [*F*_(1, 37)_ = 4.7, *p* < 0.05, and η_*p*_^2^ = 0.11], with the Cue-P3 being more positive in the MA group (3.69 μV) than in the HC group (1.85 μV). The interaction effect between group and incentive conditions was not significant [*F*_(2, 74)_ = 0.19, *p* = 0.83, and η_*p*_^2^ = 0.005] (Figures 2, **7**).

##### CNV

**Figure 3:**
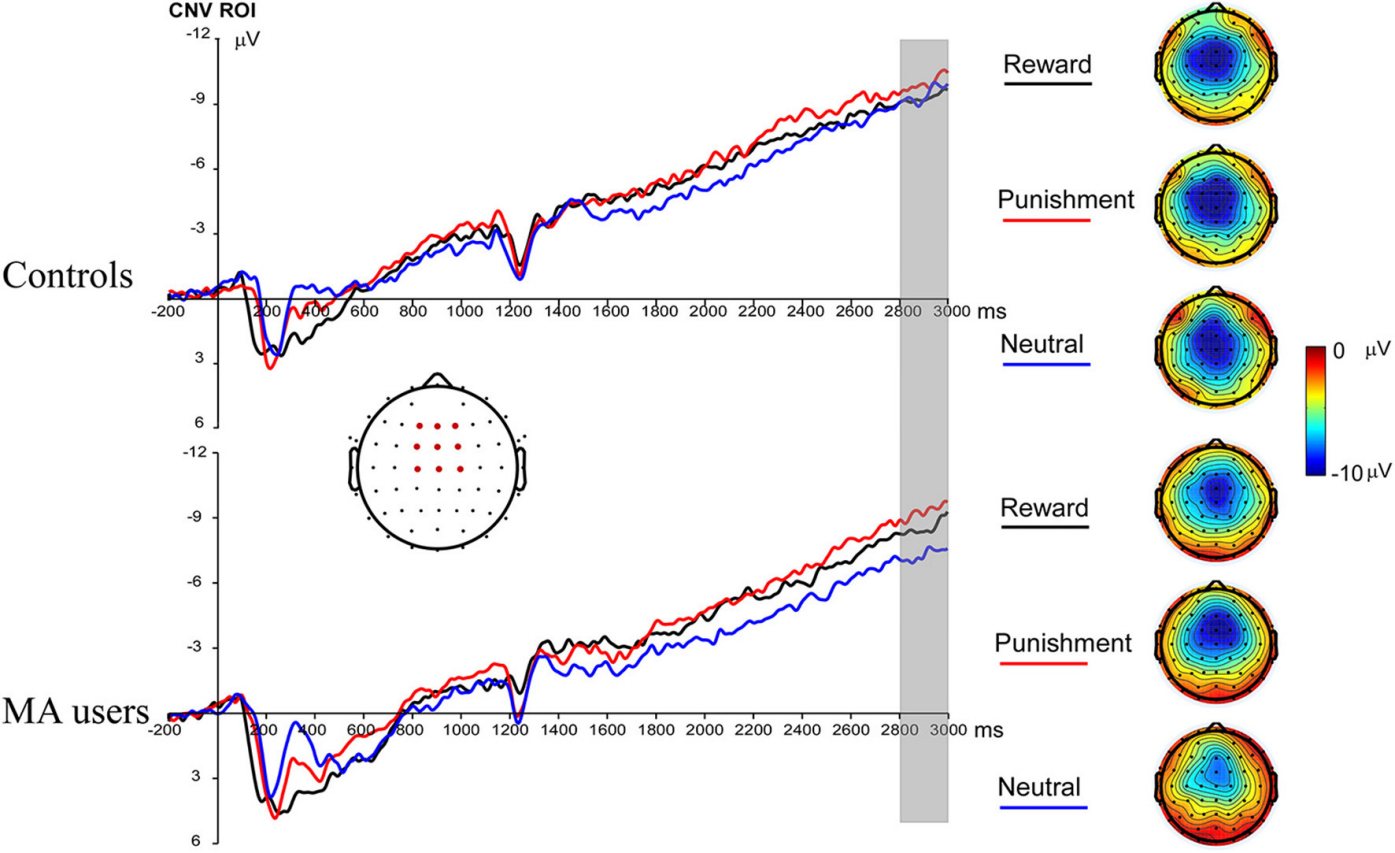
Contingent negative variation (CNV) waveforms before choice making for the MA and HC group over frontocentral ROI (F1, F2, Fz, FC1, FC2, FCz, C1, C2, and Cz) (left); topographic maps of the CNV during 2,800–3,000 ms post cue onset (right).

A 2 × 3 ANOVA was performed on the CNV data. There was no significant group effect on CNV [*F*_(1, 37)_ = 0.61, *p* = 0.44, and η_*p*_^2^ < 0.01]. Neither the incentive effect [*F*_(2, 74)_ = 2.23, *p* = 0.12, and η_*p*_^2^ = 0.06], nor the interaction effect between group and incentive conditions was significant [*F*_(2, 74)_ = 0.8, *p* = 0.45, and η_*p*_^2^ = 0.02] (Figures 3, **7**).

##### SPN

**Figure 4:**
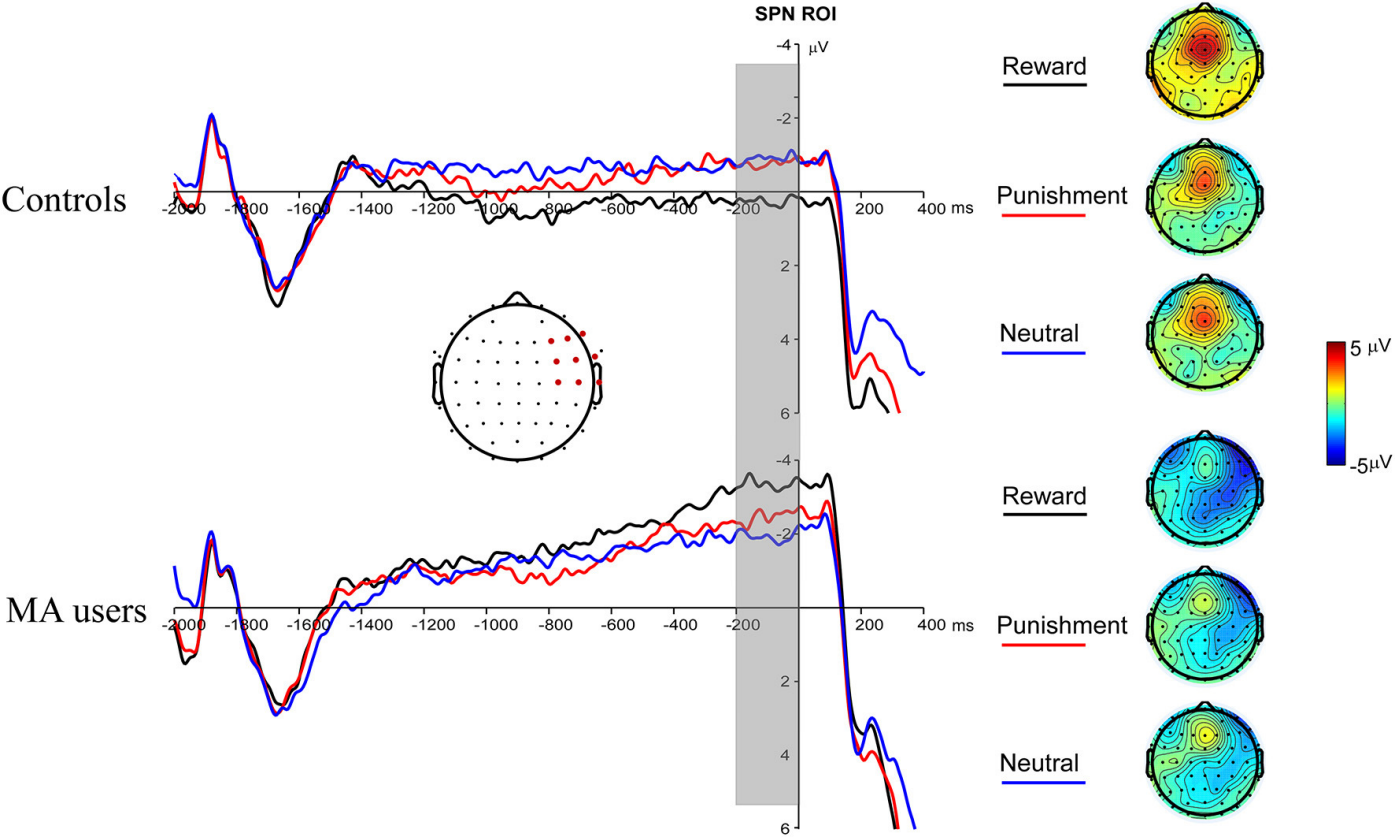
Stimulus-preceding negativity (SPN) waveforms following choice making for the MA and HC group over right frontotemporal ROI (F8, FT8, T8, F6, FC6, C6, F4, FC4, and C4) (left); topographic maps of the SPN during −200 to 0 ms before feedback onset (right).

A 2 × 3 ANOVA was performed on the SPN data. The main effect of the group was marginally significant [*F*_(1, 37)_ = 3.03, *p* = 0.09, and η_*p*_^2^ = 0.08], and the SPN in the MA group (−2.59 μV) was larger than that in the HC group (−0.47 μV). The main effect of incentive was not significant [*F*_(2, 74)_ = 0.03, *p* = 0.97]. The interaction between incentive and group was significant [*F*_(2, 74)_ = 4.31, *p* < 0.05, and η_*p*_^2^ = 0.1]. Simple analysis showed that the incentive effect was significant in the MA group, the SPN under the potential reward condition (−3.36 μV) was larger than in the neutral condition (−1.93 μV, *p* < 0.05), and no significant difference existed between potential punishment and neutral conditions. However, the incentive effect was not significant in the HC group. The SPN in potential reward condition was significantly larger in the MA group (−3.36 μV, *p* < 0.05) compared to the HC group (0.41 μV), but not in the potential punishment (*p* = 0.14, −0.68 μV in the HC group) and neutral conditions (Figures 4, **7**).

#### Consummatory ERPs

##### FRN

A 2 × 3 × 2 ANOVA was performed on the FRN data. The main effect of group was significant [*F*_(1, 37)_ = 4.6, *p* < 0.05, and η_*p*_^2^ = 0.11], and the FRN in the MA group (10.96 μV) was more positive than that in the HC group (8.6 μV). The main effect of the incentive condition was significant, [*F*_(2, 74)_ = 29.43, *p* < 0.001, and η_*p*_^2^ = 0.44], and the FRN in the potential reward (10.7 μV) and potential punishment (10.39 μV) conditions was more positive than that in the neutral condition (8.25 μV). The main effect of feedback outcome was significant [*F*_(1, 37)_ = 7.02, *p* < 0.05, and η_*p*_^2^ = 0.16], and the FRN for positive feedback (10.26 μV) was significantly higher than that for negative feedback (9.3 μV). The interaction effect of feedback and incentives was significant [*F*_(2, 74)_ = 16.53, *p* < 0.001, and η_*p*_^2^ = 0.31]. Simple analysis showed that the feedback effect was significant in the potential reward condition (*p* < 0.001, *M* = 11.82 μV following positive feedback, and *M* = 9.49 μV following negative feedback), potential punishment condition (*p* < 0.05, *M* = 9.79 μV following positive feedback, and *M* = 10.92 μV following negative feedback), and neutral condition (*p* < 0.01, *M* = 9.07 μV following positive feedback, and *M* = 7.39 μV following negative feedback). Neither the interaction effect of incentives and group [*F*_(2, 74)_ = 2.81, *p* = 0.07, and η_*p*_^2^ = 0.07], nor the interaction effect of feedback and group was significant [*F*_(1, 37)_ < 0.01, *p* = 0.99, and η_*p*_^2^ < 0.01]. The three-way effect of feedback, incentives, and group was not significant [*F*_(2, 74)_ = 0.92, *p* = 0.4, and η_*p*_^2^ = 0.02].

**Figure 5:**
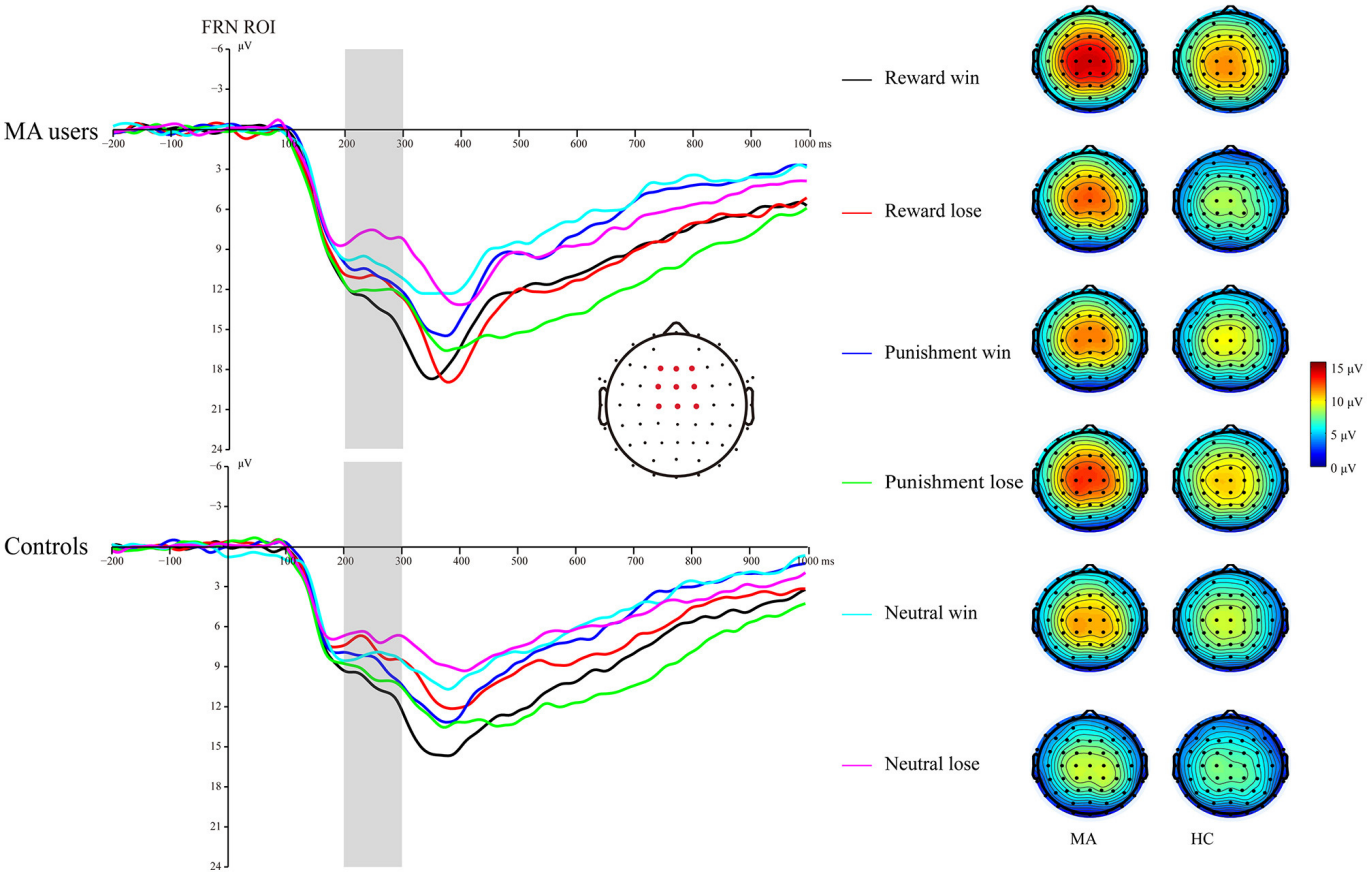
Feedback-related negativity (FRN) waveforms of win and loss for the MA group and HC group over frontocentral ROI (F1, F2, Fz, FC1, FC2, FCz, C1, C2, and Cz) post feedback (left). Topographic maps of the FRN during 200–300 ms following feedback onset (right).

We further compared the feedback effect under different incentive conditions for the MA and HC groups. Using paired *t*-test, we identified that the FRN following positive feedback was more positive than following negative feedback in the reward condition in the HC group [*t*_(19)_ = 4.44, *p* < 0.001, and *M* = 10.46 μV following positive feedback and *M* = 7.71 μV following negative feedback], but not in the MA group [*t*(_18)_ = 2.45, *p* = 0.025 > 0.008 (after Bonferroni correction), *M* = 13.25 μV following positive feedback, and *M* = 11.38 μV following negative feedback]. The FRN following positive feedback compared to negative feedback in the punishment condition was not significant in either the HC group [*t*_(19)_ = −1.58, *p* = 0.13, and *M* = 8.69 μV following positive feedback, and *M* = 9.82 μV following negative feedback] or the MA group [*t*_(18)_ = −2.37, *p* = 0.029 > 0.008 (after Bonferroni correction), *M* = 10.96 μV following positive feedback, and *M* =12.07 μV following negative feedback]. The FRN following positive feedback compared to negative feedback in the neutral condition was not significant in either the HC group [*t*_(19)_ = 1.37, *p* = 0.19, and *M* = 8.1 μV following positive feedback, and *M* = 6.82 μV following negative feedback] or the MA group [*t*_(18)_ = 2.68, *p* = 0.015 > 0.008 (after Bonferroni correction), *M* = 10.1 μV following positive feedback, and *M* =8 μV following negative feedback] (Figures 5, **7**).

##### FB-P3

A 2 × 3 × 2 ANOVA was performed on the FB-P3 data. The main effect of incentive was significant [*F*_(2, 74)_ = 26.99, *p* < 0.001, and η_*p*_^2^ = 0.42], the FB-P3 in the reward condition (15.84 μV) and punishment conditions (14.45 μV) was significantly higher than that in the neutral condition (11.57 μV, *p*s < 0.001), and the FB-P3 in the reward condition was significantly higher than that in punishment condition (*p* < 0.01). The main effect of feedback was significant [*F*_(1, 37)_ = 4.4, *p* < 0.05, and η_*p*_^2^ = 0.11], and the FB-P3 after positive feedback (14.4 μV) was higher than that of negative feedback (13.5 μV). The main effect of group was not significant [*F*_(1, 37)_ = 2.21, *p* = 0.15, and η_*p*_^2^ = 0.06]. The interaction effect of feedback and incentive was significant [*F*_(2, 74)_ = 14.25, *p* < 0.001, and η_*p*_^2^ = 0.28]. Simple analysis showed that the feedback effect was significant under the potential reward condition (*p* < 0.001, *M* = 17.66 μV following positive feedback, and *M* = 13.96 μV following negative feedback), potential punishment condition (*p* < 0.01, *M* = 13.58 μV following positive feedback, and *M* = 15.27 μV following negative feedback), but not the neutral condition (*p* = 0.36, *M* = 11.92 μV following positive feedback, and *M* = 11.15 μV following negative feedback). The interaction effect of the incentive and group was not significant [*F*_(2, 74)_ = 0.41, *p* = 0.67, and η_*p*_^2^ = 0.01]. The interaction effect of feedback and group was not significant [*F*_(2, 74)_ = 3.61, *p* = 0.07, and η_*p*_^2^ = 0.09]. The three-way interaction effect of incentive, feedback, and group was not significant [*F*_(2, 74)_ = 0.52, *p* = 0.6, and η_*p*_^2^ = 0.01].

**Figure 6:**
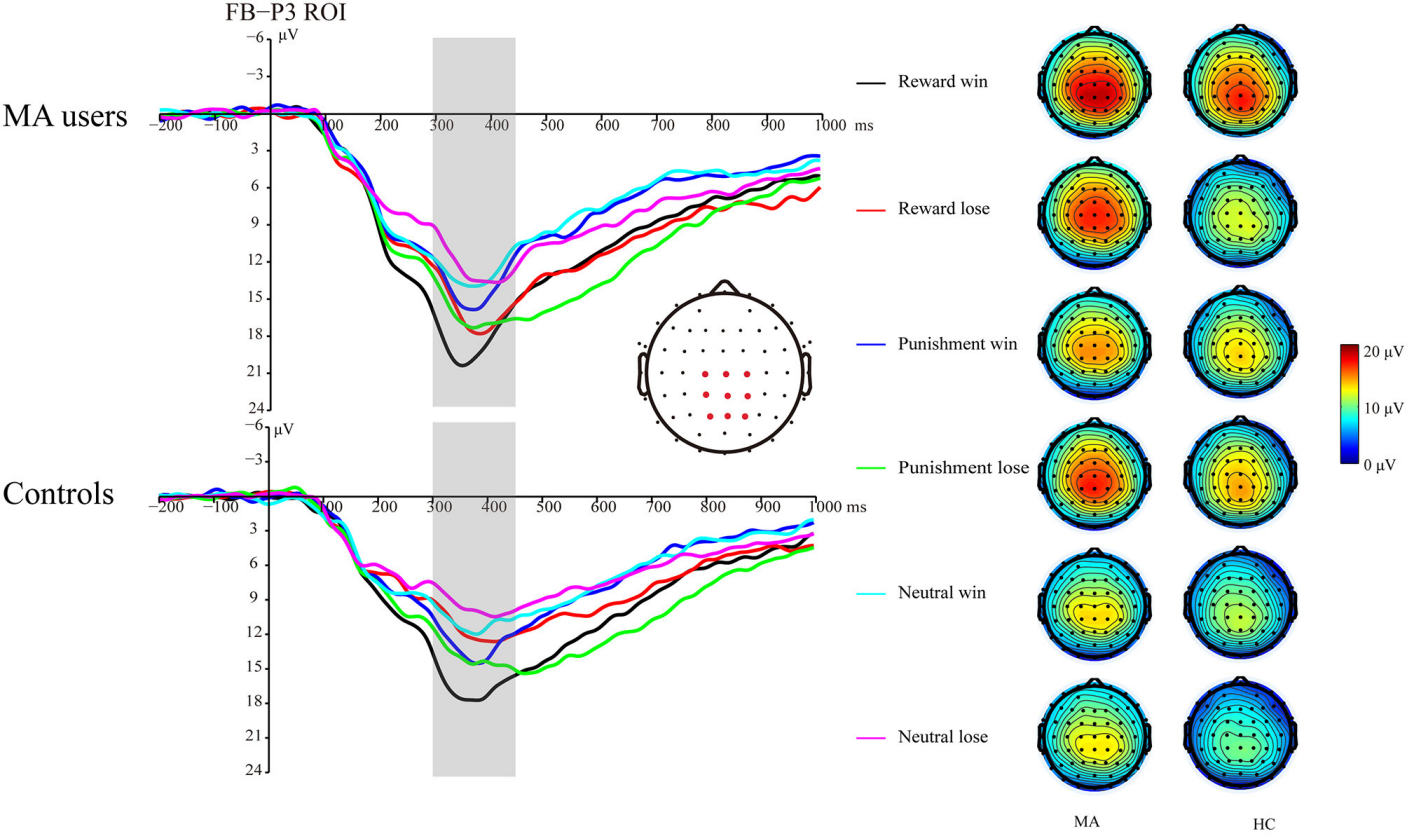
FB-P3 waveforms of win and loss for the MA and HC groups over a centroparietal ROI (C1, Cz, C2, CP1, CPz, CP2, P1, Pz, and P2) post feedback (left). Topographic maps of the FB-P3 during 300–450 ms following feedback onset (right).

**Figure 7:**
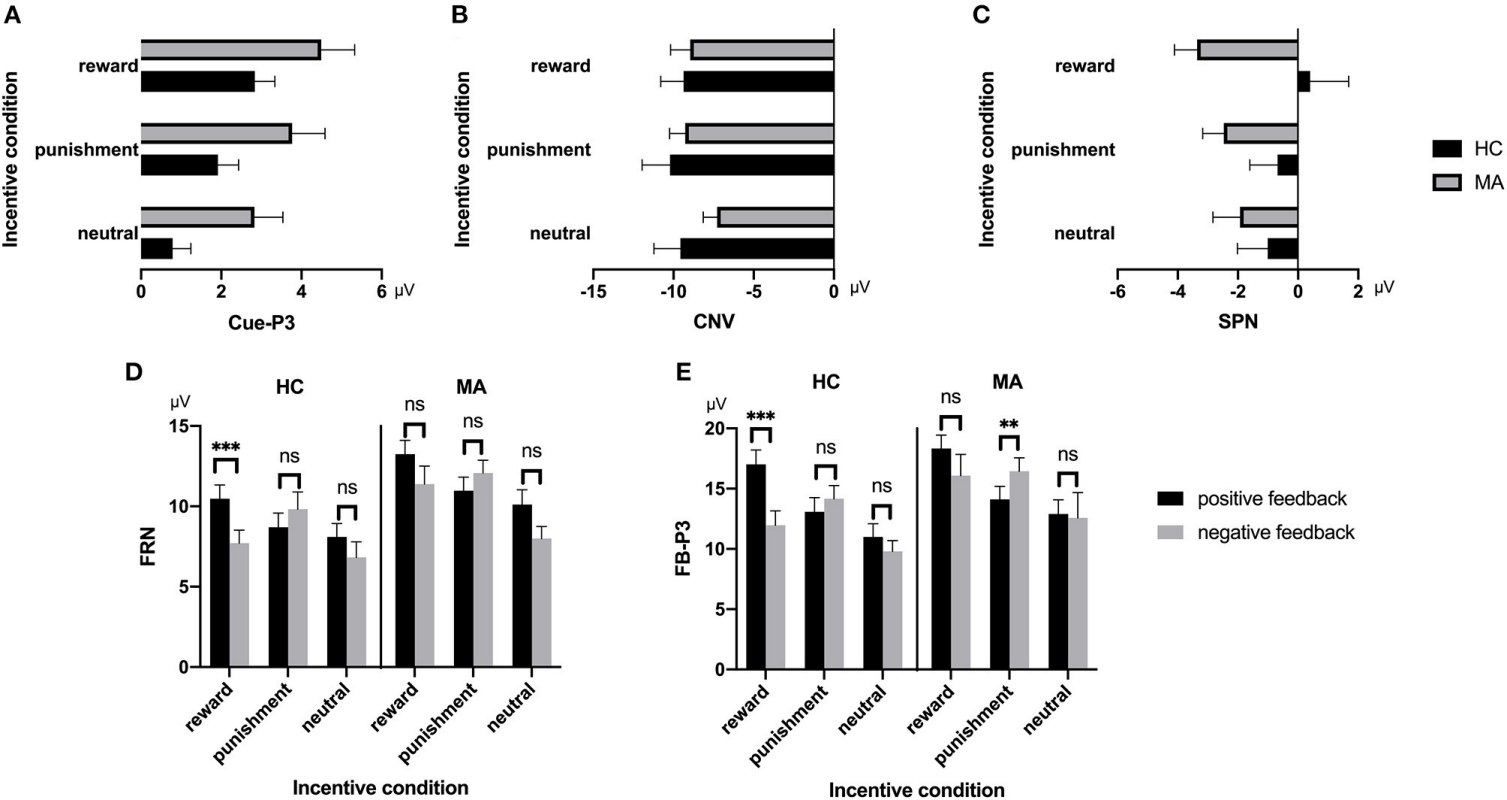
ERP component data. **(A)** Mean amplitude of Cue-P3 (during 300–450 ms after cue presentation) over the centroparietal ROI (C1, Cz, C2, CP1, CPz, CP2, P1, Pz, and P2) for MA and HC groups. **(B)** Mean amplitude of CNV (during 2,800–3,000 ms post-cue onset) before choice making over the frontocentral ROI (F1, F2, Fz, FC1, FC2, FCz, C1, C2, and Cz) for the MA and HC groups. **(C)** Mean amplitude of SPN (during −200 to 0 ms before feedback onset) following choice making over the right frontotemporal ROI (F8, FT8, T8, F6, FC6, C6, F4, FC4, and C4) for the MA and HC groups. **(D)** Mean amplitude of FRN (during 200–300 ms following feedback onset) of win and loss over the frontocentral ROI (F1, F2, Fz, FC1, FC2, FCz, C1, C2, and Cz) post feedback for the MA and HC groups. **(E)** Mean amplitude of FB-P3 (during 300–450 ms following feedback onset) of win and loss over the centroparietal ROI (C1, Cz, C2, CP1, CPz, CP2, P1, Pz, and P2) post feedback for the MA and HC groups. Standard errors are also depicted. ns, not significant, **p < 0.01, ***p < 0.001.

We further compared the feedback effect under different incentive conditions for the MA and HC groups. Using paired *t*-test, we identified that FB-P3 following positive feedback was significantly larger than that following negative feedback in the potential reward condition in the HC group [*t*_(19)_ = 5.34, *p* < 0.001, and *M* = 17.02 μV following positive feedback and *M* = 11.96 μV following negative feedback], but not the MA group [*t*_(18)_ = 1.64, *p* = 0.12, and *M* = 18.33 μV following positive feedback and *M* = 16.07 μV following negative feedback]. However, the FB-P3 following positive feedback was significantly lower than that following negative feedback in the potential punishment condition in the MA group [*t*_(18)_ = −5.19, *p* < 0.001, and *M* = 14.11 μV following positive feedback, and *M* =16.44 μV following negative feedback], but not in the HC group [*t*_(19)_ = −1.44, *p* = 0.17, and *M* = 13.07 μV following positive feedback, and *M* =14.16 μV following negative feedback]. The FB-P3 following positive feedback was similar to that following negative feedback in the neutral condition in the MA group [*t*_(18)_ = 0.23, *p* = 0.82, and *M* = 12.9 μV following positive feedback, and *M* =12.58 μV following negative feedback] and the HC group [*t*_(19)_ = 1.29, *p* = 0.21, and *M* = 10.99 μV following positive feedback, and *M* =9.8 μV following negative feedback] (Figures 6, 7).

## Discussion

In this study, we utilized the MID task to identify the electrophysiological brain responses to potential reward and punishment during the anticipatory and consummatory stages of monetary incentive processing in women with MUD and healthy controls. In particular, we determined that in the anticipatory stage of monetary incentive processing, the women with MUD have sensitive neural correlates to the potential reward cues, while in the consummatory stage of the monetary incentive processing, they have more sensitive neural correlates to the delivery of the punishment.

In line with previous research (57, 58), the current study showed that women with MUD had significantly higher scores on the subscales of the BIS and SSS compared with healthy controls, which suggests that women with MUD tend to be impulsive and sensation-seeking. Impulsivity is the core pathological characteristic of SUD (59, 60), which may arise *via* two alternative mechanisms, which are not mutually exclusive. First, a highly impulsive personality may create a vulnerability to recreational substance use when available, and second, chronic substances use can induce changes in brain function, leading to increased impulsivity.

In the current study, for both groups, the behavioral data showed that the response latency under the potential reward and potential punishment conditions was significantly faster than that under neutral conditions, but there were no differences between potential reward and punishment conditions. Similarly, the ACC under the potential reward and punishment conditions was significantly higher than that under the neutral condition, with no differences between potential reward and punishment conditions. The behavioral results of this study confirm previous MID research that the RT in monetary incentive conditions is faster than that in neutral conditions (61–63), which indicated that individuals are more motivated to secure a monetary gain or avoid a monetary loss (64). This study also identified that the RT of women with MUD was faster than that of HC. This finding supports Anderson et al.'s (65) research that links attentional bias for a monetary reward with addiction, which suggests that substance users have heightened attentional capture by stimuli associated with drug and non-drug rewards. However, we also found that under neutral conditions, the RT of the women with MUD was faster than that of the HC. A meta-analysis showed that individuals with MUD have greater deficits in reward- or impulse-related functions and social cognition, and moderate deficits in global cognition, attention, executive functions, language/verbal fluency, language learning and memory, visual memory and working memory, and related control (6). In the current study, individuals with MUD were required to undergo mandatory isolation treatment for 2 years, during which time they could not use drugs or smoke. Thus, the behavioral activation effects caused by the use of substances or cigarettes can be ruled out. Gray proposed the existence of two independent motivational systems: behavioral inhibition system (BIS) and behavioral activation system (BAS) (66, 67). The BIS is activated by conditioned signals of punishment and termination of reward. In contrast, the action of the BAS is engaged only by conditioned signals of reward and termination of punishment, which promotes approach and active avoidance behavior. A previous study found that college students' illegal substances use correlated positively with BAS and negatively with BIS personality characteristics (68). Therefore, the faster response in women with MUD in the current study is consistent with a hyper-sensitive “go” or BAS. Prolonged abstinence may have afforded an opportunity to recover whatever deficits active MA or other substance use might have done to undermine the “stop” or BIS.

The findings from this study demonstrate distinct ERP components in the anticipatory and consummatory stages of monetary incentive processing. Concerning anticipatory processes, the Cue-P3 was shown to reflect the allocation of attention to signals for monetary incentive conditions in both groups, such that amplitudes were more positive for potential reward and punishment conditions than neutral conditions. These results are consistent with previous reports (31, 32, 61, 62, 69–71) that confirmed the sensitivity of this component to the salient features of incentives. Moreover, the Cue-P3 was not sensitive to cue valence during incentive processing, in which both reward and punishment cues elicited greater Cue-P3 than neutral stimuli. The results of Cue-P3 are also congruent with a stronger motivation, which accounts for faster response latency and a higher accuracy rate in the monetary incentive conditions. The amplitude of Cue-P3 was significantly higher in the MA group than in the HC group, which indicates that MA users have an increased neural response to cues of monetary incentives than healthy controls.

Furthermore, in this study, the MA group had a greater amplitude of SPN compared with the HC group under the potential reward condition, but there were no differences in the amplitude of SPN between the MA and HC groups under the potential punishment and neutral conditions. The results of Cue-P3 and SPN in this study showed that women with MUD have increased motivation for monetary rewards. These results are consistent with the previous fMRI results indicating increased neural activity during monetary reward anticipation in individuals with alcohol dependence and gambling disorder (22, 72). However, Luijten et al. (23) indicated that individuals with substance and gambling addiction showed decreased striatal activation compared with healthy controls in a meta-analysis. Compared with previous ERP studies, these results are consistent with our previous finding that women with MUD have an increased SPN to reward anticipation in a simple gambling task (46). These results are also consistent with those of Morie et al. (40), who demonstrated that cocaine users showed amplified anticipatory responses to reward predictive cues. However, Zhao et al. (41) showed that abstinent heroin users showed neural hypoactivation during the reward anticipation stage. This differs from the findings of this study. According to reward-deficiency theory, SUD is associated with a hypodopaminergic reward system (73), which suggests reduced neural responses to non-drug rewards (74). However, substance users have also been shown to exhibit impulsive behavior, particularly involving hyperactive responses to monetary rewards (75). The results could also support the incentive-sensitization theory, which proposes that substance users are characterized by hypersensitive anticipatory reward processing (i.e., the “wanting” process) (76). The focus of sensitized “wanting” in addiction is supposed to be primarily toward drug cues, rather than non-drug rewards (77). However, a previous study further indicated that chronic exposure to substances of abuse could lead to sensitization, which enhances the pursuit of natural rewards in animals (78). Therefore, these results support the impulsivity and incentive-sensitization theories in addiction.

The incentive effect was not observed for CNV, which is consistent with previous studies adopting the MID task to an ERP design (62, 69, 70). However, this is in contrast with other studies that observed a greater CNV following reward and loss cues relative to neutral cues (32, 79). The CNV is hypothesized to consist of anticipatory attention and preparation of the movement (35). The current results suggest that although the substance users had increased anticipatory monetary incentive processing, they also had similar motor preparation for pressing the button in both the monetary incentive and neutral conditions of this study.

Regarding consummatory ERPs, the FRN is sensitive to performance evaluation and reward evaluation during feedback processing and signals greater negativity when an outcome is worse than expected (80, 81). In this study, we identified that the FRN of the negative feedback was significantly greater than that for the positive feedback under the potential reward condition in the HC group, but no feedback effect was indicated under the potential punishment condition. However, the feedback effect of the FRN was displayed in neither the reward context nor the punishment context in the MA group. Similarly, previous studies showed that FRN was more negative for negative feedback than for positive feedback in the gain or win frame, but with no difference between the positive and negative frames in the loss frame (82–84). The framing effect is a well-established phenomenon, in which most people tend to be risk-averse in the gain frame but risk-seeking in the loss frame in risky decision-making (85). The current results support the existence of frame effects in healthy controls, but not women with MUD. The MA users were not sensitive to negative feedback in the potential reward context. Previous studies found that individuals with cocaine and alcohol use disorder showed blunted sensitivity to monetary reward outcomes indexed by decreased FRN (53, 86). The present findings suggest that prolonged abstinence from stimulants in women with a history of heavy MA use does not alter this deficit, raising the possibility that low FRN may predispose a person to substance addiction. However, there are also studies that showed enhanced FRN to monetary feedback in heroin or MA users (41, 46).

In the monetary incentive consummatory stage, under the potential reward conditions, the FB-P3 of the positive feedback was significantly greater than that of the negative feedback in healthy controls, while the no feedback effect of FB-P3 existed in the MA users. The results suggest that under the potential reward condition, healthy controls were more sensitive to positive. In this study, the HC group was sensitized to positive feedback under the potential reward condition, while the MA group showed a significantly higher neural response to both the positive and negative feedback. However, the MA group was sensitive to negative feedback under the potential punishment condition, while the HC group showed a similar neural response to both positive and negative feedback. The FB-P3 is sensitive to more unexpected outcomes (87) but not sensitive to performance evaluation (88, 89). The current results suggest that the MA users are hyperactive to monetary loss under the potential punishment condition.

Similarly, one previous study identified that MA users exhibited more response in the caudate to loss outcomes than to gain outcomes (36). Another study indicated that smokers had higher academic scores from punishment feedback than non-smoking controls (90). According to early models of addiction (91), addicted individuals take drugs to alleviate or avoid aversive withdrawal syndrome. Solomon and Corbit (92) postulated that the initial effects of addictive drugs are appetitive, but these effects trigger the activation of a negative or opponent process. Solomon concluded that negative reinforcement has the most potent motivational influence on drug use. Recent researchers (8, 93) posit that a negative affect addiction stage, which involves avoidance of negative emotional after-effects of drug use, plays an important role in addiction. According to these theories, withdrawal-based learning makes drug users have a sensitive response to negative affect, which leads to drug use. In our previous study, we found that the individuals with MUD were more likely to make risky decisions following negative feedback (46). Therefore, the MA users' sensitivity to negative feedback under the potential punishment condition may be related to negative reinforcement. However, previous studies have also identified that individuals with SUD or pathological gamblers are less sensitive to punishment than healthy controls (25–27, 94). Since there are relatively few studies on the neural mechanism of addicted individuals in punishment processing, more research is required to clarify this issue.

Although our results provide some new information, some limitations still need to be considered. This study only included women with MUD, and future studies should be cautious when extending these results to male MA users. Moreover, female users were recruited from compulsory addiction rehabilitation centers, and their living environments were isolated from the outside world. Due to these limitations, current research results cannot be extended to men or individuals who do not seek treatment. Further studies are required to verify the current conclusions in other populations.

## Conclusion

Using a MID task for ERP research, this study examined the incentive processing under the reward and punishment conditions in women with MUD and healthy controls. In this study, we revealed that women with MUD are more sensitive to monetary reward anticipation and monetary punishment consummation than healthy controls. The results suggest that women with MUD have stronger expectations of generic reward and stronger response of generic harm avoidance, which could be targeted in designing interventions for women with MA use disorder.

## Data Availability Statement

The raw data supporting the conclusions of this article will be made available by the authors, without undue reservation.

## Ethics Statement

The studies involving human participants were reviewed and approved by the Ethical Review Board of the Institute of Psychology of the Chinese Academy of Science. The patients/participants provided their written informed consent to participate in this study.

## Author Contributions

SW, HW, and XL conceived and designed this study. SW and ZX designed experimental stimuli and procedures. WS and JH implemented experimental protocols and collected data. SW and WS analyzed data. SW wrote the paper. All authors contributed to the article and approved the submitted version.

## Funding

This work was supported by the National Key R&D Program of China (2016YFC0800901-Z03), the National Social Science Foundation of China (18BSH128), and in part by the Natural Science Foundation of Guangdong Province (2021A1515012509).

## Conflict of Interest

The authors declare that the research was conducted in the absence of any commercial or financial relationships that could be construed as a potential conflict of interest. The Handling Editor XW declared a shared affiliation, though no other collaboration, with one of the authors ZX at the time of the review.

## Publisher's Note

All claims expressed in this article are solely those of the authors and do not necessarily represent those of their affiliated organizations, or those of the publisher, the editors and the reviewers. Any product that may be evaluated in this article, or claim that may be made by its manufacturer, is not guaranteed or endorsed by the publisher.
